# Trichosporon asahii and Candida guilliermondii as a Source of Orbital Infection in an Immunocompromised Individual

**DOI:** 10.7759/cureus.79062

**Published:** 2025-02-15

**Authors:** David Morcos, Kevin Hardy, Anthony Makovec, Morgan Schill, John Foxworth

**Affiliations:** 1 Medical School, University of Missouri-Kansas City School of Medicine, Kansas City, USA; 2 Medical School, Louisiana State University Health Sciences Center, New Orleans, USA; 3 Internal Medicine, University of Missouri-Kansas City School of Medicine, Kansas City, USA; 4 Radiology, University Health Truman Medical Center, Kansas City, USA

**Keywords:** candida guillermondii, immunocompromised patient, peripherally inserted central catheter (picc), rhino-orbital- cerebral mucormycosis, trichosporon asahii

## Abstract

A 47-year-old male with a history of immunosuppression and recent intensive care unit admission presented with progressive orbital swelling and pain. Blood cultures grew *Trichosporon asahii* and *Candida guilliermondii*, with matching positive cultures from peripheral and central venous samples. Given his fungemia and worsening orbital involvement, induction therapy with amphotericin B and isavuconazole was initiated. Within weeks, clinical improvement was noted, prompting a transition to long-term consolidation therapy with oral posaconazole and isavuconazole. At follow-up, the patient demonstrated sustained clinical stability with no recurrence of infection. Orbital involvement from fungemia due to *T. asahii* and *C. guilliermondii* is rare, and management requires early recognition, aggressive antifungal therapy, and careful monitoring.

## Introduction

*Trichosporon* spp. is a broad genus of basidiomycetous yeasts that form arthroconidia, commonly found in soil, decomposing wood, and certain animals [1]. These organisms can colonize human mucosal surfaces, occasionally causing severe invasive disease known as trichosporonosis [1]. *T. asahii* is the most prevalent species and has been linked to endocarditis, retinitis, and skin infections, typically in immunocompromised patients [2-5]. Established risk factors include hematologic and pulmonary disorders, diabetes, ICU admission, and invasive devices [6-7]. Although consensus guidelines remain limited, posaconazole or voriconazole is often recommended for trichosporonosis, with amphotericin B and isavuconazole considered alternatives [8,9]. In the past, fluconazole, an antifungal classified within the triazole family, was suggested as a first-line treatment for infection with *Trichosporon *spp. However, due to increasing resistance reported, potentially attributed to mutations in the ERG11 gene, its use has been limited in invasive disease [10].

The *Candida guilliermondii* complex is likewise pervasive and is part of the normal human flora, yet it accounts for only a small fraction of candidemia [11-12]. It can manifest as onychomycosis, periodontitis, osteomyelitis, or endocarditis, especially in immunocompromised individuals. Risk factors include exposure to antibiotics, steroids, long-term antifungals, and intravascular catheter use [13]. Most strains remain susceptible to amphotericin B, though decreased fluconazole sensitivity and intrinsic resistance to echinocandins have been noted [14-15].

Infection with either *Trichosporon asahii* or *Candida guilliermondii* is quite rare, to the best of our knowledge, and no cases have been reported with infection of both organisms simultaneously. Here, we report a case of combined trichosporonosis and candidemia in an immunocompromised individual following treatment of rhino-orbital mucormycosis with a peripherally inserted central catheter (PICC line).

## Case presentation

**Figure 1:**
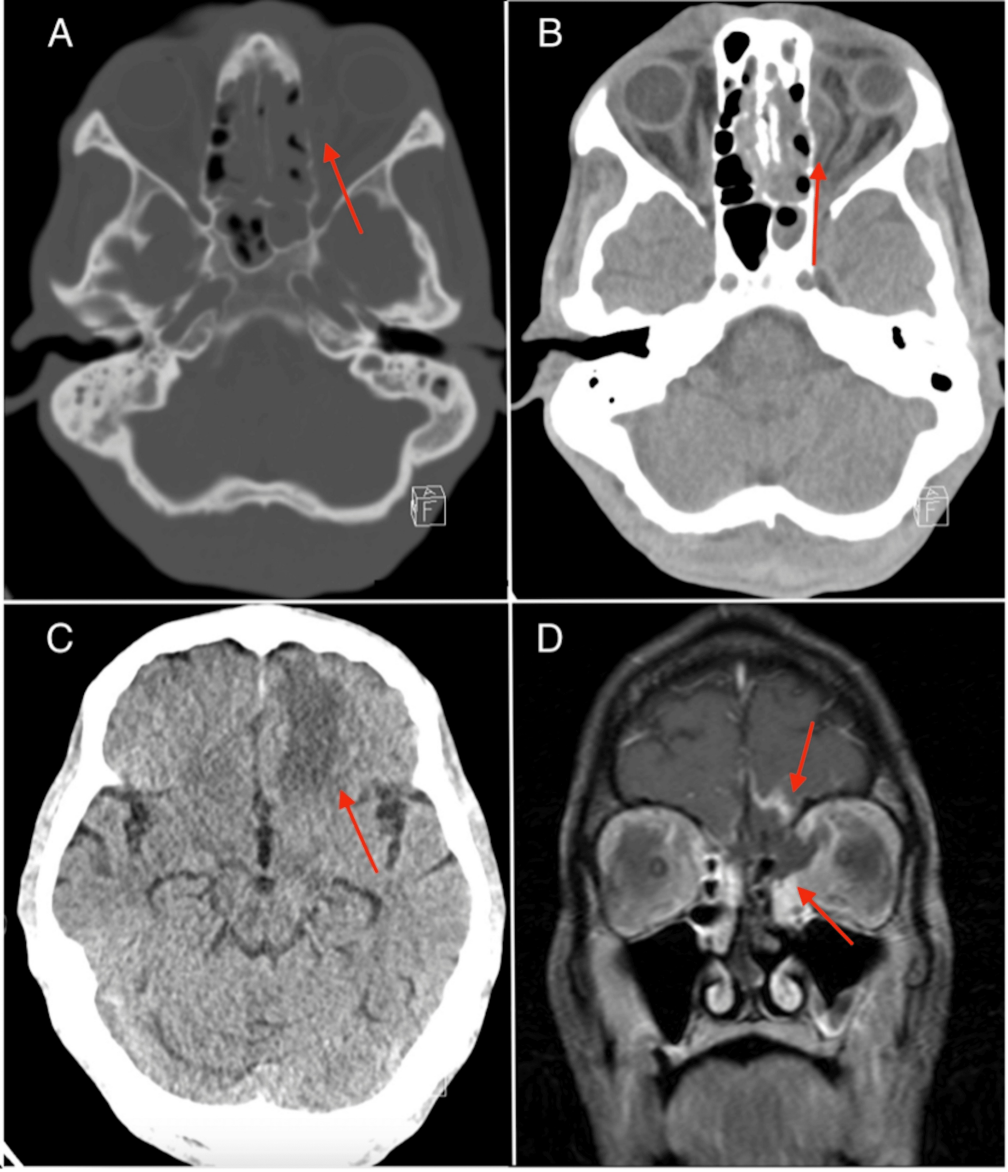
Imaging suggestive of rhino-orbital cerebral mucormycosis Axial non-contrast CT head images show [A] opacification of the ethmoid and sphenoid sinuses with osseous erosion of the left medial orbital wall, [B] trans-osseous extension into the left retro-orbital space, and [C] left greater-than-right frontal lobe parenchymal hypodensity. [d] Coronal T1-weighted fat-saturated contrast-enhanced MRI orbit shows partially non-enhancing sinonasal mucosa with disease extension into the left medial orbit and frontal lobes.

A 47-year-old male with a history of poorly controlled type 1 diabetes (with frequent hospitalizations for diabetic ketoacidosis), provoked right upper extremity deep vein thrombosis, hepatitis C, and multiple prior admissions presented with acute left-sided vision loss, ophthalmoplegia, and periorbital edema. On the initial ophthalmic evaluation of the left eye, best-corrected visual acuity was reduced to no light perception (NLP), pupillary testing revealed a mid-dilated pupil with sluggish reflexes and a relative afferent pupillary defect, and funduscopic examination demonstrated attenuated, sclerotic vessels alongside a flat, pale, ischemic retina. Imaging suggested rhino-orbital cerebral mucormycosis in the context of uncontrolled diabetes (Figure 1). 

The patient underwent surgical intervention including anterior skull base resection, bifrontal craniectomy, left orbital exenteration, left medial maxillectomy, and septectomy. Intraoperative cultures grew *Mucor* spp., *Candida albicans*, methicillin-resistant *Staphylococcus aureus*, and *Pichia kudriavzevii*. An intravenous antimicrobial regimen of amphotericin B (250 mg/day), isavuconazole (372 mg/day), ceftriaxone (2 g/day), vancomycin (15 mg/kg/q12h), and metronidazole (500 mg/q8h) was initiated. During a postoperative ophthalmologic examination, the exenteration site was noted to be clean, with no evidence of active bleeding or infection, indicating stable healing and an absence of orbital complications. After 12 days, the individual was discharged on oral amoxicillin-clavulanate (875 mg-125 mg/q12h), doxycycline (100 mg/q12h), and posaconazole (300 mg/day).

The patient returned a second time with left orbital pain and dried blood at the exenteration site. Imaging suggested an infraorbital artery bleed, but the patient declined intervention at the time. Intravenous linezolid (600 mg/q12h), daptomycin (dose adjusted by weight, once daily), and meropenem (2 g/q6h) were administered via a PICC line to prevent infection. The patient's oral antifungal was switched from posaconazole to isavuconazole (372 mg/day) indefinitely. The follow-up sinus debridement was uncomplicated, and a transthoracic echocardiogram ruled out valvular involvement. The individual was discharged to a long-term acute care facility.

Four weeks later, the patient was re-admitted for sepsis, with vitals showing severe hypotension, tachycardia, and fever (103 °F). The patient also had severe left orbital socket pain. Nasal endoscopy revealed purulent discharge, which was cultured. Intravenous meropenem (2 g/q6h), linezolid (600 mg/q12h), and micafungin (150 mg/day) were started. Staff at the long-term care facility noted a malfunction in the device delivering antimicrobials, resulting in an unknown lapse in treatment. Within 24 hours, cultures from the left eye socket, central line, and PICC line isolated both *Trichosporon asahii* and *Candida guilliermondii* (Table 1). A differential time-to-positivity of more than two hours implicated the PICC line as the source, prompting its removal. Micafungin was discontinued due to likely resistance, and intravenous amphotericin B (250 mg/day) plus isavuconazole (372 mg/day) was initiated via a peripheral line. Clinical improvement was noted, with decreased pain, reduced orbital discharge, and negative follow-up cultures.

**Table 1:** Rapid culture results from various sites during initial presentation

Site of Culture	Result	Time Difference
Orbit	1+ *Trichosporon asahii *	-
Central line	1+ Trichosporon asahii	>2 hours
Peripheral line	2+ Trichosporon asahii , 2+ Candida guilliermondii	

Final sensitivities obtained two weeks later confirmed susceptibility profiles of *T. asahii* and *C. guilliermondii *(Table 2, 3]. The regimen was then transitioned to oral amphotericin B and isavuconazole, and the patient was discharged on consolidation therapy with posaconazole (300 mg loading dose followed by 300 mg daily) and isavuconazole (372 mg daily) indefinitely to prevent recurrence.

**Table 2:** Culture susceptibility results from cultures taken from the peripherally inserted central catheter on presentation regarding Trichosporon asahii.

Antifungals tested: Trichosporon asahii	MIC	Interpretation
Fluconazole	8	N/A
Itraconazole	0.5	N/A
Amphotericin B	0.5	N/A
Anidulafungin	-	Resistant
Caspofungin	-	Resistant
Micafungin	-	Resistant
Posaconazole	0.5	N/A

**Table 3:** Culture susceptibility results from cultures taken from the peripherally inserted central catheter on presentation regarding Candida guilliermondii.

Antifungals tested: Candida guilliermondii	MIC	Interpretation
Fluconazole	8	N/A
Itraconazole	0.5	N/A
Amphotericin b	0.5	N/A
Anidulafungin	1	N/A
Caspofungin	0.5	N/A
Micafungin	0.5	N/A
Posaconazole	0.5	N/A

## Discussion

While *Trichosporon asahii *and *Candida guilliermondii* are rare causes of sepsis, special attention should be given to patients presenting with infection with these organisms given the potential for significant morbidity and mortality [16]. Our patient, similar to other patients presenting with* Trichosporon asahii* or *Candida guilliermondii*, had several risk factors for developing serious complications, including but not limited to uncontrolled type I diabetes, recent ICU stay, and a PICC line. Given the nature of infection in our patient, systemic antifungals were warranted to reduce mortality. 

Our patient’s culture susceptibility results suggested resistance of the isolated *Trichosporon* spp. to echinocandins, including anidulafungin, caspofungin, and micafungin, similar to what has been reported in the literature [9,17]. While the cultures obtained in our study included MIC values for amphotericin B and other azoles, the laboratory was unable to interpret the MIC values to determine their in vitro sensitivities (Table 2, 3). This is a quite common phenomenon for rare organisms, and ultimately led to the decision to use the current literature to guide our antimicrobial selection [8,9,14,15,17]. In our case, amphotericin B and isavuconazole proved to have good in vivo activity against both *Trichosporon asahii* and *Candida guilliermondii*, as demonstrated by marked improved clinical status and subsequent negative cultures. 

In addition, we chose to include posaconazole and isuvaconazole as adjuncts to treatment, due to their potential to reduce mortality. In a recent review, the mortality rate in Trichosporon fungemia was 63% with azole-based therapy vs. 100% in the absence of any azole drug in the therapeutic regimen [18]. This further supports our use of posaconazole and isavuconazole as part of a consolidation strategy following amphotericin B.

While several *Candida* species have been implicated in orbital infections, *Trichosporon *spp. is rarely reported, mainly manifesting as keratitis, endophthalmitis, or retinitis [4,19,20]. In most cases, *Trichosporon* infections occurred postoperatively, though endogenous infection via fungemia has also been noted. Similar to the present case, amphotericin B combined with an azole was the predominant approach. Outcomes have varied, ranging from resolution of infection with antifungals alone to more invasive interventions such as pars plana vitrectomy or capsulotomy [19,20]. In our case, a similar dual antifungal strategy adequately addressed orbital involvement caused by both *T. asahii* and *C. guilliermondii*, highlighting the potential for this combination beyond what is currently described in the literature.

## Conclusions

In conclusion, we hope to shed light on the possibility of concurrent fungemia resulting in orbital infection with both T*richosporon asahii *and *Candida guilliermondii*. By demonstrating successful treatment, as demonstrated by subsequently negative blood cultures and decreased pain in our patient, we aim to guide the management of future, comparable cases.
